# Baseline characteristics from a 3-year longitudinal study to phenotype subjects with COPD: the FOOTPRINTS study

**DOI:** 10.1186/s12931-023-02584-2

**Published:** 2023-11-17

**Authors:** James D. Crapo, Abhya Gupta, David A. Lynch, Alice M. Turner, Robert M. Mroz, Wim Janssens, Andrea Ludwig-Sengpiel, Harald Koegler, Anastasia Eleftheraki, Frank Risse, Claudia Diefenbach

**Affiliations:** 1 Department of Medicine, National Jewish Health, Denver, CO USA; 2 TA Inflammation Medicine, Boehringer Ingelheim International GmbH, Biberach an Der Riss, Germany; 3 Department of Radiology, National Jewish Health, Denver, CO USA; 4 Institute of Applied Health Research, University of Birmingham, Birmingham, UK; 5 2nd Department of Lung Diseases and Tuberculosis, Bialystok Medical University, Bialystok, Poland; 6 Department of Chronic Diseases and Metabolism (CHROMETA), Laboratory of Respiratory Diseases and Thoracic Surgery (BREATH), University Hospital Leuven, Louvain, KU Belgium; 7 KLB Gesundheitsforschung Lübeck GmbH, Lübeck, Germany; 8 TA Inflammation Medicine, Boehringer Ingelheim International GmbH, Ingelheim, Germany; 9 Department of Translational Medicine and Clinical Pharmacology, Boehringer Ingelheim Pharma GmbH & Co. KG, Biberach an Der Riss, Germany

**Keywords:** Chronic obstructive pulmonary disease, Emphysema, FOOTPRINTS^®^, Baseline characteristics, Alpha 1 antitrypsin deficiency, Biomarkers, Disease progression

## Abstract

**Background:**

FOOTPRINTS^®^ is a prospective, longitudinal, 3-year study assessing the association between biomarkers of inflammation/lung tissue destruction and chronic obstructive pulmonary disease (COPD) severity and progression in ex-smokers with mild-to-severe COPD. Here, we present baseline characteristics and select biomarkers of study subjects.

**Methods:**

The methodology of FOOTPRINTS^®^ has been published previously. The study population included ex-smokers with a range of COPD severities (Global Initiative for Chronic Obstructive Lung Disease [GOLD] stages 1–3), ex-smokers with COPD and alpha-1-antitrypsin deficiency (A1ATD) and a control group of ex-smokers without airflow limitation (EwAL). At study entry, data were collected for: demographics, disease characteristics, history of comorbidities and COPD exacerbations, symptoms, lung function and volume, exercise capacity, soluble biomarkers, and quantitative and qualitative computed tomography. Baseline data are presented with descriptive statistical comparisons for soluble biomarkers in the individual GOLD and A1ATD groups versus EwAL.

**Results:**

In total, 463 subjects were enrolled. The per-protocol set comprised 456 subjects, mostly male (64.5%). The mean (standard deviation) age was 60.7 (6.9) years. At baseline, increasing pulmonary symptoms, worse lung function, increased residual volume, reduced diffusing capacity of the lung for carbon monoxide (DLco) and greater prevalence of centrilobular emphysema were observed with increasing disease severity amongst GOLD 1–3 subjects. Subjects with A1ATD (n = 19) had similar lung function parameters to GOLD 2–3 subjects, a high residual volume comparable to GOLD 3 subjects, and similar air trapping to GOLD 2 subjects. Compared with EwAL (n = 61), subjects with A1ATD had worse lung function, increased residual volume, reduced DLco, and a greater prevalence of confluent or advanced destructive emphysema. The soluble inflammatory biomarkers white blood cell count, fibrinogen, high-sensitivity C-reactive protein and plasma surfactant protein were higher in GOLD 1–3 groups than in the EwAL group. Interleukin-6 was expressed less often in EwAL subjects compared with subjects in the GOLD and A1ATD groups. Soluble receptor for advanced glycation end product was lowest in GOLD 3 subjects, indicative of more severe emphysema.

**Conclusions:**

These findings provide context for upcoming results from FOOTPRINTS^®^, which aims to establish correlations between biomarkers and disease progression in a representative COPD population.

*Trial registration number: *NCT02719184, study start date 13/04/2016.

**Supplementary Information:**

The online version contains supplementary material available at 10.1186/s12931-023-02584-2.

## Introduction

Chronic obstructive pulmonary disease (COPD) is a progressive disease primarily of the lungs, and is characterised by persistent respiratory symptoms and airflow limitation due to airway and alveolar abnormalities. COPD is generally attributed to exposure to tobacco and/or harmful air particles [1]. Changes to the small airways in COPD, such as obstructive bronchiolitis and parenchymal destruction (emphysema), can occur alone or in combination, and the relative contributions and severity of each vary between individuals [1].

In the era of precision medicine, phenotyping patients with COPD based on one or more disease characteristics could improve our understanding of pathogenesis and disease progression, as patients do not generally all follow the same trajectory in their disease. As a result, this may allow for more targeted, individualised, and hence efficacious pharmacological and non-pharmacological management. Prospective longitudinal studies can help to assess whether soluble biomarkers (e.g., from whole blood, serum, plasma or sputum), imaging biomarkers and clinical parameters, or combinations therein, may serve as predictors of disease progression in COPD. Several studies in COPD have either completed or are underway to help address this question, including COPDGene [2], ECLIPSE [3], SPIROMICS [4, 5], COSYCONET [6], CanCOLD [7] and the British Lung Foundation early COPD study [8].

FOOTPRINTS^®^ (ClinicalTrials.gov: NCT02719184) is a 3-year study aiming to assess the association between soluble biomarkers of inflammation/lung tissue destruction, and imaging and physiological biomarkers, with COPD progression in ex-smokers with mild-to-severe COPD (as per the Global Initiative for Chronic Obstructive Lung Disease [GOLD] classification) [9]. In addition, this study includes a subgroup of 19 subjects with COPD and alpha-1-antitrypsin deficiency (A1ATD), a genetic condition associated with a higher risk of developing emphysema [10]. In A1ATD, emphysema occurs at a younger age and progresses faster as compared with COPD patients without A1ATD [11, 12].

The data from FOOTPRINTS^®^ may allow identification of different COPD phenotypes, the biomarkers that help differentiate those phenotypes, and their associated risks for emphysema progression. More detailed information on the methodology of the FOOTPRINTS^®^ study has been published previously [13]. Here, baseline demographic and clinical characteristics (lung function, soluble biomarkers, functional imaging findings and respiratory symptoms) for the study subjects are reported, as well as a descriptive statistical comparison of soluble biomarker levels in the individual GOLD groups and A1ATD group versus ex-smokers without airflow limitation (EwAL).

## Methods

### Study design

Details of the methodology for the FOOTPRINTS^®^ study (NCT02719184) have been published previously [13]. In brief, the FOOTPRINTS^®^ study is a multinational, prospective, longitudinal, observational biomarker study.

The study population included subjects with a range of COPD severities (GOLD stage 1: forced expiratory volume in 1 s [FEV_1_] ≥ 80% predicted; GOLD stage 2: FEV_1_ ≥ 50– < 80% predicted; and GOLD stage 3: FEV_1_ ≥ 30– < 50% predicted), subjects with COPD and A1ATD, and an EwAL control group. All subjects in this study should have stopped smoking at least 9 months prior to inclusion [9]. The EwAL control group was included to provide a comparison for biomarkers between subjects with and without airflow limitation while controlling for the potential effects of previous smoking.

### Inclusion and exclusion criteria

Full details on the inclusion and exclusion criteria for participation in the FOOTPRINTS^®^ study have been published previously [13]. Post-bronchodilator FEV_1_/forced vital capacity [FVC] < 70% was required for inclusion in the COPD and A1ATD groups, and post-bronchodilator FEV_1_/FVC ≥ lower limit of normal (as defined by the GOLD 2015 strategy report) was required for inclusion in the EwAL group. Subjects that were heterozygous or homozygous for the A1AT Z or S alleles were not included in the COPD GOLD groups 1–3 and EwAL group. Subjects with A1ATD were required to have a documented (prior to Visit 2) A1ATD of ZZ genotype. Patients in the COPD and A1ATD groups who were currently receiving or planned to receive A1AT augmentation therapy were excluded.

### Baseline parameters of interest

At study entry, data were collected for demographic and disease characteristics, history of comorbidities and COPD exacerbations, and soluble biomarkers in various biofluids (whole blood, serum, plasma and induced sputum). History of comorbidities or COPD exacerbations included an event/disease that had occurred at least once during the lifetime of a subject.

Data on the 6-min walk test (6MWT) were collected for all subjects and symptom questionnaires were conducted for subjects in the COPD and A1ATD groups. Pulmonary symptoms were assessed using the following questionnaires: the COPD Assessment Test (CAT), the modified Medical Research Council (mMRC) dyspnoea score, and St. George’s Respiratory Questionnaire (SGRQ). Exercise capacity was assessed via the 6MWT. Baseline quantitative and qualitative X-ray computed tomography (CT) data were collected for all subjects.

### Biomarker characterisation

Biomarkers were analysed in several biofluids at study entry and are being assessed up to Week 156. The serum/plasma biomarkers collected include, but are not limited to, neutrophil elastase-specific elastin fragment, other protease-generated neoepitopes such as cathepsin S-cleaved decorin, soluble receptor for advanced glycation end product (sRAGE), surfactant protein D (SP-D), lysyl oxidase-like 2, interleukin-6 (IL-6), high-sensitivity C-reactive protein (hs-CRP), white blood cell (WBC) count and fibrinogen.

Soluble biomarkers from sputum included biomarkers for the neutrophil-derived serine proteases neutrophil elastase, cathepsin G and proteinase 3 activity. These are not reported in this publication.

### Quantitative and qualitative CT

Chest CT scans were performed to assess lung disease, including emphysema, air trapping, small airway disease and airway wall thickness. At four time points (Week 0 [i.e., Day 1], Week 52, Week 104 and Week 156), two different scans were performed: (1) a low-dose inspiratory chest CT scan with dose modulation to assess airway walls and emphysema, and (2) an expiratory scan to assess air trapping. The inspiratory CT technique was similar to the reduced dose technique developed for Phase 3 of COPDGene [14]. To optimise assessment of non-reversible air trapping, the chest CT scan was conducted between 1 and 4 h after salbutamol (albuterol) administration.

The Fleischner criteria for CT imaging in COPD were used for visual characterisation of centrilobular and paraseptal emphysema and of airway disease [15]. Quantitative analysis of lung images was performed centrally using LungQ (Thirona B.v., Nijmegen, the Netherlands). Assessments included inspiratory and expiratory volume, emphysema, air trapping and the square root of the wall area of bronchus with an internal perimeter of 10 mm. For emphysema, analyses included lung density at the 15th percentile point of the CT histogram (PD15), adjusted lung density (ALD) and percentage of lung voxels with attenuation ≤ –950 Hounsfield Units in inspiration (LAA-950) [13]. For air trapping, the analyses included percentage of lung voxels with attenuation ≤ –856 Hounsfield Units (LAA-856) not including parametric response mapping, mean lung density during expiration and the ratio of expiratory and inspiratory volume (function residual capacity [FRC]/total lung capacity [TLC] ratio) [13].

### Statistical methods

All baseline data are presented descriptively for each of the subject subgroups, either as mean and standard deviation (SD) for normally distributed variables, median and interquartile range (IQR) for variables deviating from normality, or as number and percentage for categorical variables. To evaluate whether the continuous baseline variables showed a trend across the subject subgroups ordered by disease severity, linear regression (for normally distributed variables) or the Jonckheere-Terpstra test (for non-parametric comparisons) were conducted. For categorical variables with ≥ 2 categories, a Cochran–Armitage test for trend or a Cochran–Mantel–Haenszel test was applied, respectively. If a trend was not established, a Chi-squared test was performed to investigate associations. All soluble biomarkers were log2-transformed. Due to a high number of values below the limit of quantification, IL-6 was analysed as a categorical variable, with a lower limit of quantification equal to 3.12 ng/L used as a cut-off for categorisation. All analyses were hypothesis-generating and no correction for multiple testing was performed. Analyses were conducted using SAS v9.4.

## Results

### Subject disposition

**Fig. 1:**
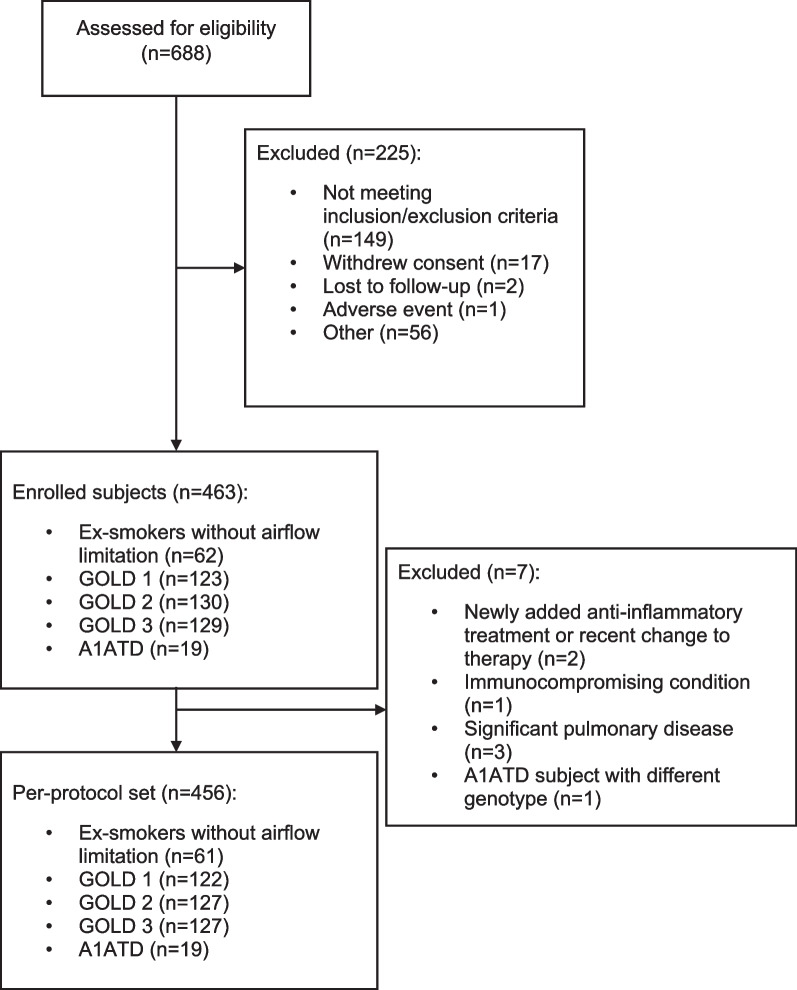
CONSORT flow diagram. *A1ATD *alpha-1-antitrypsin deficiency, GOLD Global Initiative for Chronic Obstructive Lung Disease

In total, 463 subjects were enrolled into the FOOTPRINTS^®^ study (Fig. 1). Of these, 123 subjects were categorised as GOLD stage 1, 130 as GOLD stage 2, and 129 as GOLD stage 3. Nineteen subjects had COPD and homozygous ZZ A1ATD and 62 were EwAL. Of the 19 subjects in the A1ATD group, 1 subject (5.3%) met the criteria for GOLD stage 1, 5 (26.3%) for GOLD stage 2, 12 (63.2%) for GOLD stage 3, and 1 (5.3%) for GOLD stage 4.

All demographics and analyses are based on the per-protocol set, which comprised 456 subjects (Table 1). Seven subjects were excluded from the per-protocol set due to newly added anti-inflammatory treatment or change to therapy prior to Visit 1, presenting with an immunocompromising condition, significant pulmonary disease other than COPD, or A1ATD subjects with a genotype other than ZZ.

**Table 1:** Subject demographics

	EwAL	GOLD 1	GOLD 2	GOLD 3	A1ATD
Enrolled subjects, n	62	123	130	129	19
Per-protocol subjects, n	61	122	127	127	19
Male, n (%)	38 (62.3)	85 (69.7)	76 (59.8)	79 (62.2)	16 (84.2)
Age, years	53.9 (6.8)	62.0 (6.2)	61.8 (5.4)	62.9 (5.6)	52.8 (9.0)
BMI, kg/m^2^	26.48 (2.74)	27.16 (3.43)	26.66 (4.07)	26.33 (4.32)	25.06 (3.87)
Smoking history, pack-years	32.9 (14.6)	41.5 (17.6)	46.1 (18.2)	49.2 (21.6)	25.2 (12.3)
Subjects with ≥ 1 comorbidities^a^, n (%)^b^	NA	84 (68.9)	108 (85.0)	97 (76.4)	14 (73.7)
Subjects with ≥ 3 comorbidities^a^, n (%)^b^	NA	33 (27.0)	50 (39.4)	51 (40.2)	4 (21.1)
History of COPD exacerbations, n (%)					
Yes No Missing	NA NA NA	30 (24.6) 88 (72.1) 4 (3.3)	48 (37.8) 79 (62.2) 0 (0.0)	71 (55.9) 55 (43.3) 1 (0.8)	12 (63.2) 7 (36.8) 0 (0.0)

Age, BMI and smoking history are mean (SD). A1ATD subjects are not included in the GOLD 1–3 columns

*A1ATD* alpha-1-antitrypsin deficiency, *BMI* body mass index, *COPD* chronic obstructive pulmonary disease, *EwAL* ex-smokers without airflow limitation, *GOLD* Global Initiative for Chronic Obstructive Lung Disease, *NA* not assessed, *SD* standard deviation

^a^Pneumonia, pulmonary fibrosis, chronic respiratory failure, asthma–chronic pulmonary disease overlap syndrome, obstructive sleep apnoea, bronchiectasis, pleural effusion, lung cancer, angina pectoris, ischaemic heart disease (incl. myocardial infarction), systemic arterial hypertension, hypotension, aneurysm, syncope, cardiac arrhythmia, heart failure, cerebrovascular disease, diabetes mellitus, obesity, hyper-/dyslipidaemia, cholecystitis, cholecystolithiasis, colitis, gastroesophageal reflux disease, dementia, cognitive impairment, depression, anxiety, osteoarticular disorders

^b^Data were missing for four subjects in the GOLD 1 group, one in GOLD 3, and one in the A1ATD group

### Baseline characteristics

Selected baseline characteristics are shown in Table 1. The majority of subjects were male (n = 294, 64.5%) and the mean (± SD) age was 60.7 (6.9) years. Mean (± SD) body mass index was 26.6 kg/m^2^ (3.8) and mean (± SD) smoking pack history was 43.1 pack-years (19.5). Of note, a higher proportion of subjects in the A1ATD group were male (84.2%) compared with subjects in the GOLD 1–3 groups (59.8–69.7%) and EwAL group (62.3%). The mean age was lower in the A1ATD group (52.8 ± 9.0) and EwAL group (53.9 ± 6.8) than in the GOLD 1–3 groups (61.8 ± 5.4–62.9 ± 5.6). Smoking pack history was also lower in the A1ATD group (25.2 ± 12.3) and EwAL (32.9 ± 14.6) than in the GOLD 1─3 groups (41.5 ± 17.6–49.2 ± 21.6).

As disease severity increased, the proportion of subjects with multiple comorbidities increased (Table 1); 27.0% of GOLD 1 subjects had ≥ 3 comorbidities compared with 39.4% of GOLD 2 and 40.2% of GOLD 3 subjects. The most common comorbidities (> 10% prevalence) in GOLD 1, 2 and 3 subjects were, respectively, systemic arterial hypertension (36.1%, 40.9%, 49.6%), hyper-/dyslipidaemia (20.5%, 31.5%, 29.1%), pneumonia (4.9%, 19.7%, 22.8%), gastroesophageal reflux disease (14.8%, 15.7%, 15.7%), depression (9.0%, 17.3%, 15.7%), osteoarticular disorders (10.7%, 18.1%, 14.2%), diabetes (13.1%, 13.4%, 11.0%), anxiety (6.6%, 14.2%, 14.2%) and obesity (4.9%, 12.6%, 12.6%). Further details are given in the supplementary material (Additional file 1). Of the subjects in the A1ATD group, 21.1% had ≥ 3 comorbidities, but, as noted above, this was a younger population, with subjects being on average 10 years younger.

The percentage of prior COPD exacerbations (defined as an exacerbation that had occurred at least once during the lifetime of a subject) increased with increasing disease severity (as per GOLD classification): 24.6%, 37.8% and 55.9% of GOLD 1, GOLD 2 and GOLD 3 subjects, respectively, had experienced a previous COPD exacerbation. Overall, 40.8% of COPD subjects had a documented COPD exacerbation in their medical history.

Further information on baseline characteristics are available in Additional file 1.

### Symptom questionnaires and 6MWT

Symptom questionnaires were conducted for subjects in the GOLD 1–3 and A1ATD groups, but not for the EwAL group. As shown in Table 2, increasing disease severity was associated with an increase in symptoms. Symptom scores in the A1ATD group were intermediate between those of the GOLD 1 and 3 groups. Increasing disease severity was associated with increases in the mean CAT score (GOLD 1: 11.2 [SD 5.7]; GOLD 2: 14.7 [SD 6.2]; GOLD 3: 16.6 [SD 7.2]; A1ATD: 13.4 [SD 7.4], P < 0.0001), the proportion of subjects with a CAT score ≥ 10 (GOLD 1: 62.3%; GOLD 2: 78.0%; GOLD 3: 79.5%; A1ATD: 63.2%, P = 0.0335), the proportion of subjects with an mMRC dyspnoea score ≥ 2 (GOLD 1: 11.5%; GOLD 2: 32.3%; GOLD 3: 60.6%; A1ATD: 47.4%, P < 0.0001) and the proportion of subjects with an SGRQ score ≥ 25 (GOLD 1: 50.8%; GOLD 2: 69.3%; GOLD 3: 83.5%; A1ATD: 73.7%, P < 0.0001).

**Table 2:** Symptom questionnaires and 6-min walk test

	EwAL	GOLD 1	GOLD 2	GOLD 3	A1ATD	P-value
Per-protocol subjects, n	61	122	127	127	19	
CAT total score	NA	11.2 (5.7)	14.7 (6.2)	16.6 (7.2)	13.4 (7.4)	< 0.0001
CAT total score, n (%)						
< 10 ≥ 10	NA NA	45 (36.9) 76 (62.3)	28 (22.0) 99 (78.0)	26 (20.5) 101 (79.5)	7 (36.8) 12 (63.2)	0.0335*
mMRC dyspnoea score, n (%)						
< 2 ≥ 2	NA NA	107 (87.7) 14 (11.5)	86 (67.7) 41 (32.3)	50 (39.4) 77 (60.6)	10 (52.6) 9 (47.4)	< 0.0001*
SGRQ total score, n (%)	NA	25.2 (14.4)	34.7 (15.4)	43.0 (16.5)	37.7 (22.3)	< 0.0001
< 25 ≥ 25 Missing	NA NA	58 (47.5) 62 (50.8) 2 (1.6)	38 (29.9) 88 (69.3) 1 (0.8)	20 (15.7) 106 (83.5) 1 (0.8)	5 (26.3) 14 (73.7) 0 (0.0)	< 0.0001*
SGRQ activity score	NA	35.6 (20.2)	48.9 (20.0)	60.1 (18.3)	54.8 (28.1)	< 0.0001
SGRQ impacts score	NA	17.0 (13.3)	24.0 (16.4)	32.8 (17.9)	27.2 (21.8)	< 0.0001
SGRQ symptoms score	NA	33.3 (19.4)	42.8 (19.1)	44.9 (22.5)	40.2 (22.5)	0.0020
Total distance covered in 6 MWT, m	573.1 (100.3)	489.3 (91.7)	458.0 (101.3)	403.8 (121.7)	508.6 (114.4)	< 0.0001

All data are presented as mean (SD) unless noted otherwise

*6MWT* 6-min walk test, *A1ATD* alpha-1-antitrypsin deficiency, *CAT* COPD Assessment Test, *EwAL* ex-smokers without airflow limitation, *GOLD* GOLD, Global Initiative for Chronic Obstructive Lung Disease, *mMRC* modified Medical Research Council, *NA* not assessed, *SD* standard deviation, *SGRQ* St. George’s Respiratory Questionnaire

*Cochran–Armitage test for trend. A linear regression test was used for other variables

Increasing disease severity was associated with a reduction in distance covered in the 6MWT (GOLD 1: 489.3 m [SD 91.7 m]; GOLD 2: 458.0 m [SD 101.3 m]; GOLD 3: 403.8 m [SD 121.7 m], P < 0.0001). The 6MWT distance for subjects with A1ATD (508.6 m [SD 114.4 m]) was numerically higher than in any of the GOLD groups, but lower than in the EwAL group (573.1 m [SD 100.3 m]).

### Lung function and lung volume

#### Airflow limitation

**Fig. 2:**
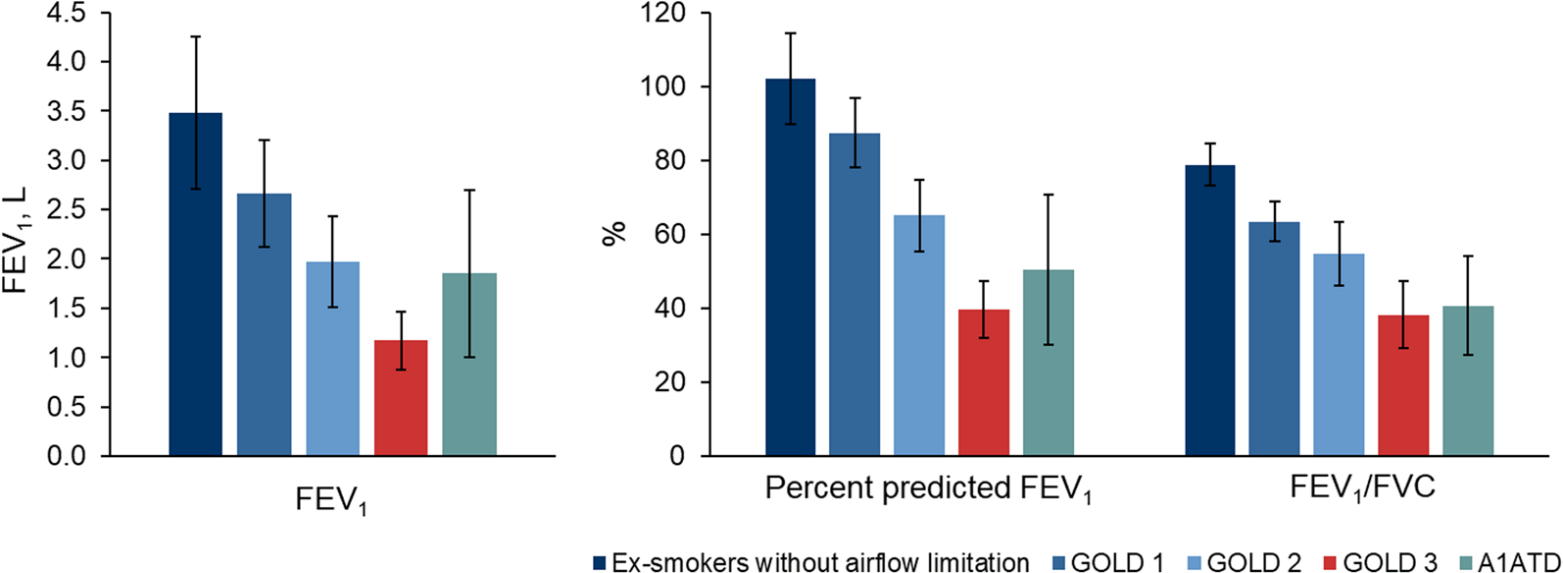
Lung function parameters. *A1ATD *alpha-1-antitrypsin deficiency, *FEV*_*1*_ forced expiratory volume in 1 s, *FVC *forced vital capacity*, GOLD *Global Initiative for Chronic Obstructive Lung Disease

At baseline, patients with the greatest disease severity (as defined by the GOLD 1–4 classification system) had the poorest lung function (Table 3; Fig. 2). Patients with COPD and A1ATD also had worse lung function than EwAL. Lung function parameters for the A1ATD group were similar to the GOLD stage 2/3 groups, with the exception of FVC.

**Table 3:** Post-bronchodilator lung function and lung volume measurements

	EwAL	GOLD 1	GOLD 2	GOLD 3	A1ATD	P-value
Per-protocol subjects, n	61	122	127	127	19	
Airflow limitation						
FEV_1_, L	3.48 (0.77)	2.66 (0.54)	1.97 (0.46)	1.17 (0.29)	1.85 (0.85)	< 0.0001
% predicted FEV_1_	102.1 (12.2)	87.4 (9.4)	65.0 (9.7)	39.6 (7.6)	50.4 (20.3)	< 0.0001
FEV_1_/FVC, %	78.8 (5.7)	63.4 (5.4)	54.6 (8.6)	38.2 (9.0)	40.6 (13.4)	< 0.0001
FVC, L	4.42 (0.97)	4.22 (0.85)	3.66 (0.90)	3.16 (0.89)	4.54 (1.38)	< 0.0001
% predicted FVC, %	102.2 (10.9)	107.7 (13.2)	93.8 (13.5)	82.6 (15.1)	96.9 (17.6)	< 0.0001
Body plethysmography						
TLC, L	6.81 (1.27)	6.70 (1.29)	6.60 (1.44)	7.02 (1.58)	8.16 (1.53)	0.0217
RV, L	2.27 (0.54)	2.51 (0.66)	2.91 (0.96)	3.64 (1.02)	3.63 (0.81)	< 0.0001
% predicted RV, %	106.5 (21.3)	111.5 (26.0)	129.0 (38.0)	161.8 (44.4)	163.0 (27.2)	< 0.0001
DL_CO_						
DL_CO_, mmol/min/kPa						
Mean (SD)	11.36 (7.66)	13.34 (8.64)	9.36 (6.52)	7.16 (5.14)	5.19 (2.02)	< 0.0001*
Median	8.77	9.28	6.75	5.18	4.78	
Range (Q1–Q3)	4.41	12.83	7.69	5.59	2.34	
% predicted DL_CO_	89.2 (16.1)	79.8 (21.3)	63.4 (18.1)	51.5 (17.5)	51.1 (12.7)	< 0.0001
DL_CO_/VA, mmol/min/kPa						
Mean (SD)	1.88 (1.20)	2.25 (1.36)	1.79 (1.19)	1.61 (1.20)	0.83 (0.19)	< 0.0001*
Median	1.38	1.48	1.23	1.04	0.85	
Range (Q1–Q3)	0.43	2.39	1.62	1.83	0.09	

All values are mean (SD)

*A1ATD* alpha-1-antitrypsin deficiency, *DL*_*CO*_ diffusing capacity of the lung for carbon monoxide, *EwAL* ex-smokers without airflow limitation, *FEV*_*1*_ forced expiratory volume in 1 s, *FVC* forced vital capacity, *GOLD* Global Initiative for Chronic Obstructive Lung Disease, *kPa* kilopascal, *mmol* millimole, *RV* residual volume, *SD* standard deviation, *TLC* total lung capacity, *VA* alveolar volume

*Jonckheere-Terpstra test for trend. A linear regression test was used for other variables

Across GOLD groups 1–3, mean FEV_1_ was highest in GOLD 1 (2.66 L [SD 0.54 L]) and lowest in GOLD 3 subjects (1.17 L [SD 0.29 L]). Mean FEV_1_ was lower in GOLD groups 1–3 and subjects with A1ATD (1.85 L [SD 0.85 L]) than in EwAL (3.48 L [SD 0.77 L]). Mean percent predicted FEV_1_ was 87.4% [SD 9.4%] in GOLD 1 subjects and decreased with increasing disease severity to 39.6% [SD 7.6%] in GOLD 3 subjects (P < 0.0001). The mean percent predicted FEV_1_ was 50.4% (SD 20.3%) in subjects with A1ATD and 102.1% (SD 12.2%) in the EwAL group. Mean FVC also decreased with increasing disease severity (GOLD 1: 4.22 L [SD 0.85 L]; GOLD 2: 3.66 L [SD 0.90 L]; GOLD 3: 3.16 L [SD 0.89 L], P < 0.0001), and, overall, was highest in subjects with A1ATD (4.54 L [SD 1.38 L]. The EwAL control group had a mean FVC of 4.42 L (SD 0.97 L). The mean FEV_1_/FVC ratio was 63.4% (SD 5.4%) in GOLD 1 subjects and reduced with increasing disease severity to 38.2% (SD 9.0%) in GOLD 3 subjects (P < 0.0001). Subjects with A1ATD had a mean FEV_1_/FVC ratio of 40.6% (SD 13.4%) and the EwAL control group FEV_1_/FVC ratio was 78.8% (SD 5.7%).

#### Plethysmography

Table 3 shows post-bronchodilator lung function and lung volume measurements at baseline. Increasing disease severity was associated with increasing mean residual volume (GOLD 1: 2.51 L [SD 0.66 L]; GOLD 2: 2.91 L [SD 0.96 L]; GOLD 3: 3.64 L [SD 1.02 L], P < 0.0001). Residual volume was high in subjects with A1ATD (3.63 L [SD 0.81 L]) and comparable with GOLD 3 COPD subjects, whereas it was lowest in EwAL (2.27 L [SD 0.54 L]).

TLC values generally increased with increasing disease severity (GOLD 1: 6.70 [SD 1.29]; GOLD 2: 6.60 [SD 1.44]; GOLD 3: 7.02 [1.58], P = 0.0217). The highest mean TLC was observed in the A1ATD group (8.16 L [SD 1.53 L]), whereas TLC for EwAL was 6.81 L (SD 1.27 L).

### Additional lung function tests

Increasing disease severity and A1ATD were associated with reduced diffusing capacity of the lung for carbon monoxide (DL_CO_), (P < 0.0001) (Table 3). The mean percent predicted DL_CO_ was 79.8% (SD 21.3%) in GOLD 1 subjects, 63.4% (SD 18.1%) in GOLD 2 subjects, 51.5% (SD 17.5%) in GOLD 3, subjects and 51.1% (SD 12.7%) in subjects with A1ATD.

The mean DL_CO_/alveolar volume ratio was highest in GOLD 1 subjects (2.25 mmol/min/kPa [SD 1.36 mmol/min/kPa]) and decreased with disease severity (GOLD 3: 1.61 mmol/min/kPa [SD 1.20 mmol/min/kPa]). Overall, mean DL_CO_/alveolar volume was lowest in the A1ATD group (0.83 mmol/min/kPa [SD 0.19 mmol/min/kPa] vs 1.88 mmol/min/kPa [SD 1.20 mmol/min/kPa] in EwAL). Further DL_CO_ parameters are available in Additional file 1*.*

### Soluble biomarkers

**Fig. 3:**
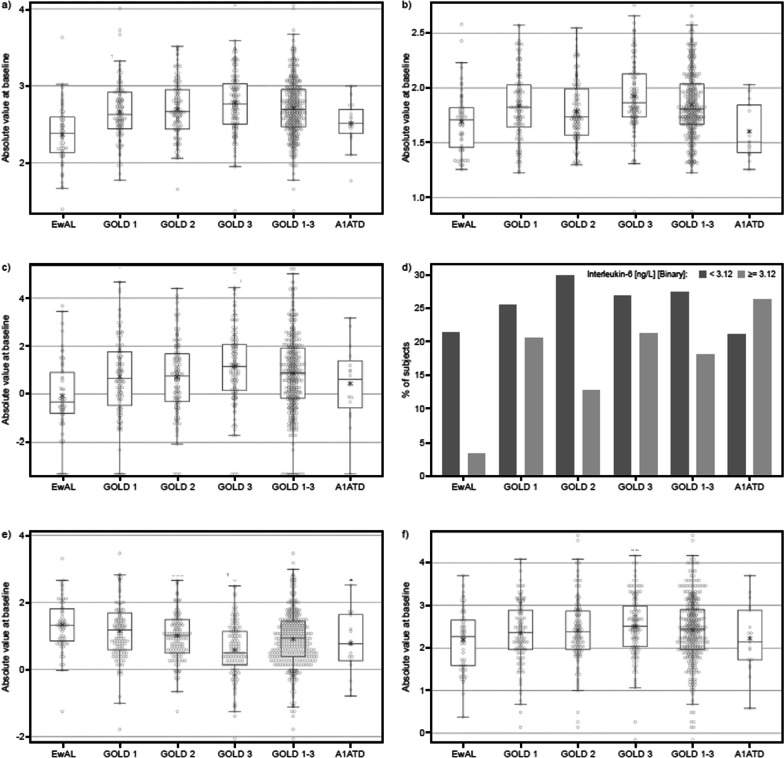
Distribution of inflammatory biomarkers at baseline by diagnosis group: **a** WBC, **b** fibrinogen, **c** hs-CRP, **d** IL-6, **e** sRAGE, **f** plasma SP-D. All biomarker values (except for IL-6) are log_2_ transformed. WBC, sRAGE: P ≤ 0.0001; hs-CRP: P = 0.0002; plasma SP-D: P = 0.0194; fibrinogen: P = 0.0249, linear regression for subject groups ordered by disease severity (Kruskal–Wallis test used for fibrinogen). No association was observed between positive expression of IL-6 and subject groups (chi-square test, P = 0.0701). *A1ATD* alpha-1-antitrypsin deficiency, *Abs* absolute, *EwAL* ex-smokers without airflow limitation, *GOLD* Global Initiative for Chronic Obstructive Lung Disease, *hs-CRP* high-sensitivity C-reactive protein, *IL-6* interleukin 6, *SP-D* surfactant protein D, *sRAGE* soluble receptor for advanced glycation end product, *WBC* white blood count

For the majority of biomarkers presented in Fig. 3, the mean expression levels or concentration increased with disease severity. A linear increase in the level of WBC (P < 0.0001), fibrinogen (P = 0.0249), hsCRP (P = 0.0002) and plasma SP-D (P = 0.0194) was observed whereas sRAGE levels linearly decreased with disease severity (P < 0.0001), indicative of more severe emphysema. In all GOLD groups (GOLD 1–3), levels of WBC, fibrinogen, hs-CRP and plasma SP-D were numerically higher compared with the EwAL group. Further soluble biomarker parameters are available in Additional file 1.

No association was detected between IL-6 positive expression and disease severity (P = 0.0701). IL-6 was expressed less often in the EwAL subjects compared with GOLD 1–3 and A1ATD subjects, of whom 13%–26% expressed IL-6 (Fig. 3d).

### Qualitative CT data

**Fig. 4:**
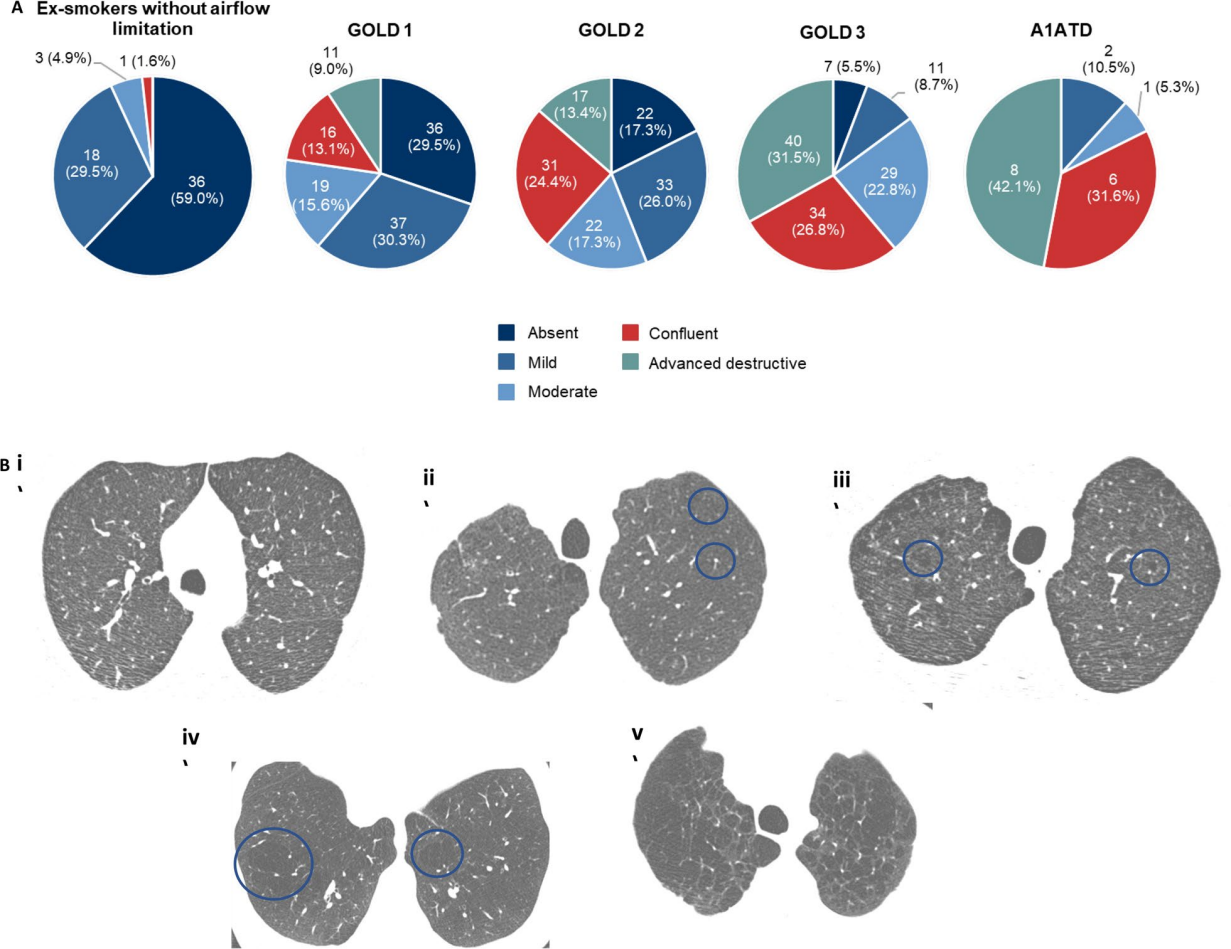
Centrilobular emphysema on CT (**A**) and CT images showing typical emphysema findings for each category i) absent, ii) mild, iii) moderate, iv) confluent, v) advanced destructive (**B**). Percentages do not total 100% due to missing data (missing: EwAL, 3.3%; GOLD 1, 2.5%; GOLD 2, 1.6%; GOLD 3, 3.9%; A1ATD, 10.5%)

Increasing disease severity was associated with a greater prevalence of centrilobular emphysema (Cochran–Mantel–Haenszel test, P < 0.0001) (Fig. 4). Emphysema was present in all subjects in the A1ATD group, and in 41.0% of the EwAL group. Among GOLD subjects, the prevalence of advanced destructive emphysema was higher in GOLD 3 (GOLD 1: 9.0%; GOLD 2: 13.4%; GOLD 3: 31.5%), as well as the prevalence of confluent emphysema (GOLD 1: 13.1%; GOLD 2: 24.4%; GOLD 3: 26.8%). The Fleischner classification does not provide a separate category for panlobular emphysema [15], which would be graded as either confluent or advanced destructive; these findings were present in 14 (82%) of 17 subjects with A1ATD.

### Quantitative CT data

When assessing lung volumes by quantitative CT, a linear increase in expiratory volume and a linear decrease in PD15 and ALD were observed with increasing disease severity (P < 0.0001 for all comparisons) (Table 4). Mean expiratory volume increased from 2.83 L (SD 0.67 L) in EwAL subjects to 4.78 L (SD 1.24 L) in GOLD 3 subjects. PD15 decreased from 91.2 g/L (SD 19.4 g/L) in EwAL subjects to 47.8 g/L (SD 24.9 g/L) in GOLD 3 subjects. PD15 was 44.4 g/L (SD 21.1 g/L) in A1ATD subjects. Similarly, ALD decreased from 88.50 g/L (SD 13.90 g/L) in EwAL subjects to 54.8 g/L (SD 23.1 g/L) in GOLD 3 subjects. ALD was 50.9 g/L (SD 18.2 g/L) in A1ATD subjects. A linear increase was also established for inspiratory volume with increasing disease severity (EwAL: 5.80 L [SD 1.30 L]; GOLD 1: 6.42 L [SD 1.28 L]; GOLD 2: 6.31 L [SD 1.35 L]; GOLD 3: 6.63 L [SD 1.45 L], P < 0.0001). Subjects in the A1ATD group had higher mean inspiratory (8.07 L [SD 1.73 L]) and expiratory (5.33 L [SD 1.51 L]) volumes than subjects in the GOLD 1–3 groups and the EwAL group.

**Table 4:** Qualitative and quantitative CT scans

	EwAL	GOLD 1	GOLD 2	GOLD 3	A1ATD	P-value
Per-protocol subjects, n	61	122	127	127	19	
Qualitative CT						
Centrilobular emphysema, N (%)						< 0.0001*
Absent Mild Moderate Confluent Advanced destructive Missing	36 (59.0) 18 (29.5) 3 (4.9) 1 (1.6) 0 (0.0) 2 (3.3)	36 (29.5) 37 (30.3) 19 (15.6) 16 (13.1) 11 (9.0) 3 (2.5)	22 (17.3) 33 (26.0) 22 (17.3) 31 (24.4) 17 (13.4) 2 (1.6)	7 (5.5) 11 (8.7) 29 (22.8) 34 (26.8) 40 (31.5) 5 (3.9)	0 (0.0) 2 (10.5) 1 (5.3) 6 (31.6) 8 (42.1) 2 (10.5)	
Quantitative CT						
Inspiratory volume, L	5.80 (1.30)	6.42 (1.28)	6.31 (1.35)	6.63 (1.45)	8.07 (1.73)	< 0.0001
Expiratory volume, L	2.83 (0.67)	3.65 (1.01)	4.01 (1.14)	4.78 (1.24)	5.33 (1.51)	< 0.0001
PD15, g/L	91.2 (19.4)	75.1 (19.0)	66.2 (22.2)	47.8 (24.9)	44.4 (21.1)	< 0.0001
ALD, g/L	88.5 (13.9)	86.2 (21.4)	72.4 (21.7)	54.8 (23.1)	50.9 (18.2)	< 0.0001
LAA-950, %	3.2 (2.8)	7.4 (7.2)	12.7 (9.9)	21.3 (12.3)	26.4 (12.0)	
LAA-856, %	7.5 (8.4)	23.0 (14.0)	36.3 (17.5)	53.5 (17.2)	51.3 (19.4)	
Pi10, mm	1.8 (0.4)	2.2 (0.5)	2.5 (0.5)	2.6 (0.4)	2.0 (0.4)	< 0.0001
MLD in expiration	− 689.1 (44.6)	− 743.0 (43.5)	− 776.8 (43.9)	− 822.3 (40.5)	− 808.4 (62.1)	< 0.0001
FRC/TLC ratio	0.5 (0.1)	0.6 (0.1)	0.6 (0.1)	0.7 (0.1)	0.7 (0.1)	< 0.0001

All values are mean (± SD)

*ALD* adjusted lung density, *A1ATD* alpha-1-antitrypsin deficiency, *CT* computed tomography, *EwAL* ex-smokers without airflow limitation, *FRC* functional residual capacity, *GOLD* Global Initiative for Chronic Obstructive Lung Disease, *LAA-856* lung voxels with attenuation ≤ –856 Hounsfield Units, *LAA-950* lung voxels with attenuation ≤ –950 Hounsfield Units, *MLD* mean lung density, *PD15* lung density at the 15th percentile of the CT histogram, *Pi10* square root of wall area of bronchus with internal perimeter of 10 mm, *SD* standard deviation, *TLC* total lung capacity

^*^Cochran–Mantel–Haenszel test. A linear regression test was used for all other variables

The mean FRC/TLC ratio, a measure of air trapping, was 0.570 (SD 0.118) in GOLD 1 subjects versus 0.722 (SD 0.097) in GOLD 3 subjects. Ex-smoker controls had the lowest FRC/TLC ratio (0.499 [SD 0.112]). Values for GOLD 2 subjects (0.629 [SD 0.102]) and A1ATD subjects (0.653 [SD 0.121]) were between the values for GOLD 1 and GOLD 3 subjects.

## Discussion

The longitudinal observational FOOTPRINTS^®^ study aims to identify biomarkers that are associated with COPD progression, in particular emphysema progression, over a 3-year period. Results from this study are expected to help address an unmet need to better understand different COPD phenotypes and their associated biomarkers. This could allow the use of biomarkers for diagnosis, to identify high-risk patients and to guide treatment options in a more targeted, precision-based manner. COPD diagnosis and treatment requires more than assessment of lung function. Biomarkers may help to better understand the biology, prognosis and treatment options suitable for different COPD phenotypes. In a study by Lowe et al. [2], for example, CT scans identified that 41% of the “healthy” EwAL subjects had centrilobular emphysema, indicating that emphysema progression had already occurred, although it was not possible to detect by traditional spirometry.

The baseline demographics and clinical characteristics of the FOOTPRINTS^®^ study subjects, including lung function parameters, soluble biomarkers, qualitative and quantitative CT, and symptoms, are provided here. These data allowed us to compare characteristics between subjects grouped by COPD severity (GOLD stages 1–3), subjects with A1ATD and EwAL subjects.

As expected, at baseline, increasing COPD severity was associated with worsening lung function, increased expiratory lung volume and a reduction in DL_CO_. Compared with EwAL, A1ATD was also associated with worsening lung function, increased lung volumes and a reduction in DL_CO_. Of note, TLC was highest in the A1ATD group compared with the other groups; however, this could be a result of the higher proportion of male and younger subjects in the A1ATD group. Levels of soluble inflammatory biomarkers such as WBC count, fibrinogen, hs-CRP and plasma SP-D increased linearly by disease severity, with higher levels observed in the GOLD 3 group. Previous studies have demonstrated increases in the expression of these inflammatory biomarkers to be associated with the progression of COPD [16, 17]. Of note, levels of fibrinogen were numerically higher in the GOLD 1 group than in the GOLD 2 group. Compared with EwAL, mean biomarker values for the A1ATD group were generally higher, except for fibrinogen, which was slightly lower for the A1ATD group. IL-6 levels were numerically lower in the EwAL group compared with GOLD 1–3 and A1ATD subjects.

Levels of sRAGE were highest in EwAL subjects and decreased linearly with increasing disease severity, with lowest values observed in the GOLD 3 group, indicative of more severe emphysema. This is in keeping with previous studies, which have suggested lower blood sRAGE to be associated with more severe airflow obstruction and emphysema [18]. Similarly, in the A1ATD group, levels of sRAGE were numerically lower than in the EwAL group, again indicative of more severe emphysema.

When assessing lung density by quantitative CT, we noted a clear and expected decrease in PD15 with increasing COPD severity, as has been shown in previous studies [19]. There was also an increase in expiratory and inspiratory volume with increasing disease severity. As disease severity increased, the presence of centrilobular emphysema increased (as assessed by qualitative CT), as did the presence of advanced destructive emphysema. As expected, subjects in the A1ATD group had the highest prevalence of confluent or advanced destructive emphysema, which presumably corresponds to panlobular emphysema (panlobular emphysema is not separately classified in the Fleischner system) [15]. Although the EwAL group did not have airflow limitation (as defined by the 2015 GOLD strategy report [9]), centrilobular emphysema was present in 36% of subjects, indicating that emphysema progression had already occurred in many of these subjects, likely a result of their 33 pack-year smoking history. Several other studies have demonstrated a substantial proportion of smokers without airflow limitation to have CT evidence of structural changes such as emphysema and airway wall thickening [2, 20, 21]. For example, in a cohort of over 4000 current and former smokers from the COPDGene study who did not meet spirometric criteria for COPD, a significant number (24%; 72 of 300) had evidence of emphysema [20]. In a study by Tan and colleagues [7] comparing ever-smokers with and without COPD against never-smokers with and without COPD using the radiological methodology of Barr et al. [22], emphysema was found in 12.8% and 10.2% in never-smokers with and without a diagnosis of COPD, compared with 54.8% and 26.0% in ever-smokers with and without a diagnosis of COPD, respectively. Thus, emphysema is strongly related to smoking history. Further analyses of the COPDGene study data suggest that a substantial proportion of smokers who do not meet the criteria for COPD, but have respiratory symptoms and imaging abnormalities, are at significant risk of death and spirometric disease progression [2]. This result highlights the importance of finding appropriate markers to identify subjects early who are at risk of disease progression. Given the high proportion of EwAL subjects who had centrilobular emphysema in our study (41%), this raises the question of whether this group could be considered an early disease group rather than a true control group.

As expected, as disease severity increased, symptoms tended to worsen. Values for subjects in the A1ATD group were generally between those in the GOLD 2 and 3 groups. This may not be surprising, given that the majority of the A1ATD subjects met the criteria for COPD GOLD 3 (n = 12, 63.2%) or GOLD 2 (n = 5, 26.3%). Our study also found that mMRC dyspnoea score (< 2 and ≥ 2) and SGRQ total score (< 25 and ≥ 25) were better discriminators for the COPD GOLD 1–3 groups than CAT score (< 10 and ≥ 10), demonstrating a more consistent numerical trend across the groups.

In addition to the FOOTPRINTS^®^ study, there are a number of longitudinal studies that are ongoing or completed in patients with COPD, including COPDGene [2], ECLIPSE [3], SPIROMICS [4, 5], COSYCONET [6], CanCOLD [7] and the British Lung Foundation early COPD study (Table 5) [8]. Comparatively, FOOTPRINTS^®^ has a number of strengths. The inclusion of the A1ATD subpopulation is unique to the study and provides information on an important but rarely studied subpopulation of patients; these patients present with earlier onset and faster progression of emphysema [12]. Further, at the time FOOTPRINTS^®^ was designed and began, no other study had presented an association between biomarkers of extracellular matrix degradation (in this case, sRAGE) and emphysema. Since then, this area has garnered the interest of several other studies [18, 23–25]. Another distinguishing factor is the method for visual assessment of emphysema used in our study; this has been conducted according to the statement by the Fleischner Society [15]. The Fleischner method has been shown to be complementary to quantitative CT assessment and to provide incremental information regarding the likelihood of disease progression, lung cancer [26] and mortality [27, 28].

**Table 5:** Summary of ongoing or completed longitudinal observational COPD studies

Observational study	Population under study	Key characteristics		Key outcomes	Duration of study (number of visits post-screening)
		COPD subjects	Control subjects		
FOOTPRINTS [13]	382 patients with COPD: • GOLD 1: 123 • GOLD 2: 130 • GOLD 3: 129 19 patients with COPD and A1ATD 62 EwAL limitation	Age (years): • 40–70 • COPD and A1ATD: 30–70 COPD & A1ATD smoking history ≥ 10 pack-years Subjects with asthma excluded	• EwAL • Smoking history ≥ 20 pack-years • FEV_1_ ≥ 80% predicted • FEV_1_/FVC ≥ LLN Post DL_CO_ ≥ 70% predicted at Visit 1	• Lung function • CT imaging • Symptom questionnaires • 6MWT • Soluble biomarkers (serum, plasma, and sputum)	3 years (5)
COPDGene [2, 29]	> 10,000 current smokers (with or without COPD) and non-smoker subjects across full spectrum of COPD disease severities	• Aged 45–80 years • ≥ 10 pack-year smoking history • Included subjects with asthma	• Non-smokers and smokers without COPD aged 45–80 years • ≥ 10 pack-year smoking history except non-smokers • Included subjects with asthma	• Lung function testing • CT imaging • Medical history • SGRQ • 6MWT • Genomic analysis, serum and plasma samples for proteomics	10 years (2)
ECLIPSE [3]	• > 2000 COPD subjects with GOLD 2–4 COPD • 343 smoking and 223 non-smoking control subjects	• Aged 40–75 years • ≥ 10 pack-year smoking history • Baseline post-bronchodilator FEV_1_ of < 80% predicted and FEV_1_/FVC ≤ 0.7	• Aged 40–75 years • Baseline post-bronchodilator FEV_1_ of > 85% predicted and FEV_1_/FVC of > 0.7 • Current or ex-smokers with a smoking history ≥ 10 pack-years (smoking controls) or non-smokers with a smoking history of < 1 pack-year	• Lung function testing • Chest CT • Biomarker measurement (blood, sputum, urine and exhaled breath condensate) • Health outcomes, body impedance and resting oxygen saturation • Symptom questionnaires • 6MWT	3 years (8)
SPIROMICS [5]	> 3200 participants in four strata: 1. Non-smokers 2. Smokers without airflow obstruction 3. Smokers with mild/moderate COPD 4. Smokers with severe COPD	Aged 40–80 years • > 20 pack-year smoking history • FEV_1_/FVC < 0.7 • FEV_1_ > 50% predicted for mild/moderate COPD and < 50% predicted for severe	Aged 40–80 years • < 1 pack-year smoking history for non-smokers • > 20 pack-year smoking history for smokers without airflow limitation • FEV_1_/FVC > 0.7 • FVC > LLN	• Lung function testing • CT imaging • 6MWT • Biomarker measurement (blood and sputum) • Symptom questionnaires • Medical history	3 years (3)
COSYCONET [6] (NCT01245933)	• 2291 patients across COPD GOLD stages 1–4 • 206 patients with GOLD “0”^a^	• Aged 40–90 years • 8%/35%/32%/9% GOLD 1–4 • 24%/68%/8% currently smoking/ex-smokers/never-smokers • Mean of 40–50 pack-years for currently smoking and ex-smokers	No control population in this study	• Lung function testing • Chest CT and MRI^b^ • 6MWT • Body impedance • Symptom and health questionnaires • Biomarker measurement (blood and urine)	9 years (7)
CanCOLD [7]	• 2295 never-smokers • 2598 ever-smokers	• Aged ≥ 40 years • > 20 pack-year smoking history in ever-smokers • Never-smokers accounted for 29% of COPD patients	No control population in this study	• Lung function testing • Chest CT • Respiratory symptoms and exacerbations	4 years (visits not listed)
UK-based British Lung Foundation early COPD [8]	• Pilot sample of 102 smokers at risk of developing clinical COPD (1000 aim to be recruited)	• Aged 30–45 years • > 10 pack-years smoking history • No/mild airflow limitation	No control population in this study	• Lung function testing • Chest CT	Unknown

*6MWT* 6-min walk test, *A1ATD* alpha-1-antitrypsin deficiency, *COPD* chronic obstructive pulmonary disease, *CT* computed tomography, *DL*_*CO*_ diffusing capacity of the lung for carbon monoxide, *EwAL* ex-smokers without airflow limitation, *FEV*_*1*_ forced expiratory volume in 1 s, *FVC* forced vital capacity, *GOLD* Global Initiative for Chronic Obstructive Lung Disease, *LLN* lower limit of normal, *MRI* magnetic resonance imaging, *SGRQ* St. George’s Respiratory Questionnaire

^a^FEV_1_/FVC > 0.7 and either (i) having a doctor diagnosis of chronic bronchitis and/or (ii) indicating a severity of cough of at least 3 in the respective COPD Assessment Test item and/or (iii) indicating a severity of phlegm of at least 3 in the respective COPD Assessment Test item

^b^Sub-cohort of 602 patients, observed for 2.5 years (NCT02629432). A follow-up study is ongoing in 370 patients (NCT03591562)

## Conclusions

The FOOTPRINTS^®^ study is investigating biomarkers associated with emphysema over a 3-year period to increase the understanding of COPD patient phenotypes, pathogenesis and disease progression. The data reported here document the baseline disease characteristics and biomarkers of subjects in our study, highlighting the substantial burden of COPD and A1ATD. In addition to the baseline biomarker data presented, future analyses will be conducted on sputum biomarkers. Importantly, these findings provide important context to precede the upcoming results from the 3-year study, which aim to establish key correlations between disease progression and biomarkers over time in a representative COPD population.

### Supplementary Information

**Additional file 1**: **Table S1**. Disease history (for categories with ≥5% in any group). **Table S2**. Soluble biomarkers in whole blood and qualitative CT scan data. **Table S3**. DL_CO_ parameters.

## Data Availability

The datasets used and/or analysed during the current study are available from the corresponding author on reasonable request.

## Abbreviations

6MWT: 6-Minute walk test
A1ATD: Alpha-1-antitrypsin deficiency
ALD: Adjusted lung density
CAT: COPD Assessment Test
COPD: Chronic obstructive pulmonary disease
CT: Computed tomography
DL_CO_: Diffusing capacity of the lung for carbon monoxide
EwAL: Ex-smokers without airflow limitation
FEV_1_: Forced expiratory volume in 1 s
FRC: Function residual capacity
FVC: Forced vital capacity
GOLD: Global Initiative for Chronic Obstructive Lung Disease
hs-CRP: High-sensitivity C-reactive protein
IL-6: Interleukin-6
LAA-856: Lung voxels with attenuation ≤ – 856 Hounsfield Units
LAA-950: Lung voxels with attenuation ≤ – 950 Hounsfield Units
mMRC: Modified Medical Research Council
PD15: 15th percentile point of the CT histogram
SGRQ: St. George’s Respiratory Questionnaire
SD: Standard deviation
SP-D: Surfactant protein D
sRAGE: Soluble receptor for advanced glycation end product
TLC: Total lung capacity
WBC: White blood cell
