# Supplementary material for: Baseline characteristics from a 3-year longitudinal study to phenotype subjects with COPD: the FOOTPRINTS study
Source: Respir Res. 2023 Nov 17;24:290. doi: 10.1186/s12931-023-02584-2 (PMC10656819; doi:10.1186/s12931-023-02584-2)
Supplement: Supplementary file 1 — Additional file 1: Table S1. Disease history (for categories with ≥5% in any group). Table S2. Soluble biomarkers in whole blood and qualitative CT scan data. Table S3. DLCO parameters. [file 12931_2023_2584_MOESM1_ESM.docx]

# Additional file 1

**Baseline characteristics from a 3-year longitudinal study to phenotype subjects with COPD: the FOOTPRINTS study**

James D. Crapo, Abhya Gupta, David A. Lynch, Alice M. Turner, Robert M. Mroz, Wim Janssens, Andrea Ludwig-Sengpiel, Harald Koegler, Anastasia Eleftheraki, Frank Risse, Claudia Diefenbach

**Table S1.** Disease history (for categories with ≥5% in any group)

|  | **GOLD 1** | **GOLD 2** | **GOLD 3** | **A1ATD** | **Total** |
| --- | --- | --- | --- | --- | --- |
| **Pneumonia, n (%)** |  |  |  |  |  |
| No | 112 (91.8) | 102 (80.3) | 97 (76.4) | 14 (73.7) | 325 (82.3) |
| Yes | 6 (4.9) | 25 (19.7) | 29 (22.8) | 4 (21.1) | 64 (16.2) |
| Missing | 4 (3.3) | 0 (0) | 1 (0.8) | 1 (5.3) | 6 (1.5) |
| **Obstructive sleep apnoea, n (%)** |  |  |  |  |  |
| No | 109 (89.3) | 112 (88.2) | 119 (93.7) | 18 (94.7) | 358 (90.6) |
| Yes | 9 (7.4) | 14 (11.0) | 7 (5.5) | 0 (0) | 30 (7.6) |
| Missing | 4 (3.3) | 1 (0.8) | 1 (0.8) | 1 (5.3) | 7 (1.8) |
| **Bronchiectasis, n (%)** |  |  |  |  |  |
| No | 117 (95.9) | 125 (98.4) | 123 (96.9) | 17 (89.5) | 382 (96.7) |
| Yes | 1 (0.8) | 2 (1.6) | 3 (2.4) | 1 (5.3) | 7 (1.8) |
| Missing | 4 (3.3) | 0 (0) | 1 (0.8) | 1 (5.3) | 6 (1.5) |
| **COPD exacerbation, n (%)** |  |  |  |  |  |
| No | 88 (72.1) | 79 (62.2) | 55 (43.3) | 7 (36.8) | 229 (58.0) |
| Yes | 30 (24.6) | 48 (37.8) | 71 (55.9) | 12 (63.2) | 161 (40.8) |
| Missing | 4 (3.3) | 0 (0) | 1 (0.8) | 0 (0) | 5 (1.3) |
| **Ischaemic heart disease including myocardial infarction, n (%)** |  |  |  |  |  |
| No | 111 (91.0) | 111 (87.4) | 118 (92.9) | 18 (94.7) | 358 (90.6) |
| Yes | 7 (5.7) | 16 (12.6) | 8 (6.3) | 0 (0) | 31 (7.8) |
| Missing | 4 (3.3) | 0 (0) | 1 (0.8) | 1 (5.3) | 6 (1.5) |
| **Systemic arterial hypertension, n (%)** |  |  |  |  |  |
| No | 74 (60.7) | 75 (59.1) | 63 (49.6) | 17 (89.5) | 229 (58.0) |
| Yes | 44 (36.1) | 52 (40.9) | 63 (49.6) | 1 (5.3) | 160 (40.5) |
| Missing | 4 (3.3) | 0 (0) | 1 (0.8) | 1 (5.3) | 6 (1.5) |
| **Aneurysm, n (%)** |  |  |  |  |  |
| No | 115 (94.3) | 125 (98.4) | 124 (97.6) | 16 (84.2) | 380 (96.2) |
| Yes | 3 (2.5) | 2 (1.6) | 2 (1.6) | 2 (10.5) | 9 (2.3) |
| Missing | 4 (3.3) | 0 (0) | 1 (0.8) | 1 (5.3) | 6 (1.5) |
| **Cardiac arrhythmia, n (%)** |  |  |  |  |  |
| No | 112 (91.8) | 119 (93.7) | 122 (96.1) | 17 (89.5) | 370 (93.7) |
| Yes | 6 (4.9) | 8 (6.3) | 4 (3.1) | 1 (5.3) | 19 (4.8) |
| Missing | 4 (3.3) | 0 (0) | 1 (0.8) | 1 (5.3) | 6 (1.5) |
| **Cerebrovascular disease, n (%)** |  |  |  |  |  |
| No | 118 (96.7) | 124 (97.6) | 122 (96.1) | 17 (89.5) | 381 (96.5) |
| Yes | 0 (0) | 3 (2.4) | 3 (2.4) | 1 (5.3) | 7 (1.8) |
| Missing | 4 (3.3) | 0 (0) | 2 (1.6) | 1 (5.3) | 7 (1.8) |
| **Diabetes mellitus, n (%)** |  |  |  |  |  |
| No | 102 (83.6) | 110 (86.6) | 111 (87.4) | 16 (84.2) | 339 (85.8) |
| Yes | 16 (13.1) | 17 (13.4) | 14 (11.0) | 2 (10.5) | 49 (12.4) |
| Missing | 4 (3.3) | 0 (0) | 2 (1.6) | 1 (5.3) | 7 (1.8) |
| **Obesity, n (%)** |  |  |  |  |  |
| No | 112 (91.8) | 111 (87.4) | 109 (85.8) | 18 (94.7) | 350 (88.6) |
| Yes | 6 (4.9) | 16 (12.6) | 16 (12.6) | 0 (0) | 38 (9.6) |
| Missing | 4 (3.3) | 0 (0) | 2 (1.6) | 1 (5.3) | 7 (1.8) |
| **Hyper-/dyslipidaemia, n (%)** |  |  |  |  |  |
| No | 93 (76.2) | 87 (68.5) | 88 (69.3) | 16 (84.2) | 284 (71.9) |
| Yes | 25 (20.5) | 40 (31.5) | 37 (29.1) | 2 (10.5) | 104 (26.3) |
| Missing | 4 (3.3) | 0 (0) | 2 (1.6) | 1 (5.3) | 7 (1.8) |
| **Cholecystolithiasis, n (%)** |  |  |  |  |  |
| No | 111 (91.0) | 122 (96.1) | 122 (96.1) | 18 (94.7) | 373 (94.4) |
| Yes | 7 (5.7) | 5 (3.9) | 3 (2.4) | 0 (0) | 15 (3.8) |
| Missing | 4 (3.3) | 0 (0) | 2 (1.6) | 1 (5.3) | 7 (1.8) |
| **GERD, n (%)** |  |  |  |  |  |
| No | 100 (82.0) | 107 (84.3) | 105 (82.7) | 16 (84.2) | 328 (83.0) |
| Yes | 18 (14.8) | 20 (15.7) | 20 (15.7) | 2 (10.5) | 60 (15.2) |
| Missing | 4 (3.3) | 0 (0) | 2 (1.6) | 1 (5.3) | 7 (1.8) |
| **Cognitive impairment, n (%)** |  |  |  |  |  |
| No | 118 (96.7) | 127 (100.0) | 125 (98.4) | 17 (89.5) | 387 (98.0) |
| Yes | 0 (0) | 0 (0) | 0 (0) | 1 (5.3) | 1 (0.3) |
| Missing | 4 (3.3) | 0 (0) | 2 (1.6) | 1 (5.3) | 7 (1.8) |
| **Depression, n (%)** |  |  |  |  |  |
| No | 107 (87.7) | 105 (82.7) | 105 (82.7) | 13 (68.4) | 330 (83.5) |
| Yes | 11 (9.0) | 22 (17.3) | 20 (15.7) | 5 (26.3) | 58 (14.7) |
| Missing | 4 (3.3) | 0 (0) | 2 (1.6) | 1 (5.3) | 7 (1.8) |
| **Anxiety, n (%)** |  |  |  |  |  |
| No | 110 (90.2) | 108 (85.0) | 107 (84.3) | 14 (73.7) | 339 (85.8) |
| Yes | 8 (6.6) | 18 (14.2) | 18 (14.2) | 4 (21.1) | 48 (12.2) |
| Missing | 4 (3.3) | 1 (0.8) | 2 (1.6) | 1 (5.3) | 8 (2.0) |
| **Osteoarticular disorders, n (%)** |  |  |  |  |  |
| No | 105 (86.1) | 104 (81.9) | 107 (84.3) | 14 (73.7) | 330 (83.5) |
| Yes | 13 (10.7) | 23 (18.1) | 18 (14.2) | 4 (21.1) | 58 (14.7) |
| Missing | 4 (3.3) | 0 (0) | 2 (1.6) | 1 (5.3) | 7 (1.8) |
| **Other (at discretion of investigator), n (%)** |  |  |  |  |  |
| No | 94 (77.0) | 95 (74.8) | 93 (73.2) | 15 (78.9) | 297 (75.2) |
| Yes | 24 (19.7) | 32 (25.2) | 31 (24.4) | 3 (15.8) | 90 (22.8) |
| Missing | 4 (3.3) | 0 (0) | 3 (2.4) | 1 (5.3) | 8 (2.0) |

*A1ATD* alpha-1-antitrypsin deficiency, *COPD*, chronic obstructive pulmonary disease, *GERD* gastroesophageal Reflux Disease, *GOLD* Global Initiative for Chronic Obstructive Lung Disease.

**Table S2.** Soluble biomarkers in whole blood and qualitative CT scan data

|  | **EwAL** | **GOLD 1** | **GOLD 2** | **GOLD 3** | **A1ATD** |
| --- | --- | --- | --- | --- | --- |
| **Soluble biomarkers in whole blood, mean (SD)** |  |  |  |  |  |
| Neutrophils, 10^9^/L | 3.064 (1.199) | 4.023 (1.484)  P<0.0001 | 3.943 (1.221)  P<0.0001 | 4.539 (1.437)  P<0.0001 | 3.296 (0.924)  P=0.0879 |
| Eosinophils, 10^9^/L | 0.164 (0.090) | 0.190 (0.113)  P=0.1742 | 0.211 (0.124)  P=0.0310 | 0.200 (0.147)  P=0.4953 | 0.206 (0.092)  P=0.2637 |
| Lymphocytes, 10^9^/L | 1.719 (0.575) | 1.914 (0.618)  P=0.0213 | 1.997 (0.680)  P=0.0058 | 1.980 (1.127)  P=0.0305 | 1.756 (0.518)  P=0.3619 |
| Monocytes, 10^9^/L | 0.352 (0.113) | 0.432 (0.159)  P=0.0010 | 0.440 (0.141)  P<0.0001 | 0.470 (0.151)  P<0.0001 | 0.365 (0.106)  P=0.2043 |
| Basophils, 10^9^/L | 0.047 (0.029) | 0.052 (0.028)  P=0.2434 | 0.061 (0.038)  P=0.0751 | 0.066 (0.044)  P=0.0158 | 0.056 (0.026)  P=0.1165 |
| **Qualitative CT scan, n (%)** |  |  |  |  |  |
| Non-emphysematous air trapping |  |  |  |  |  |
| Absent | 26 (42.6) | 76 (62.3) | 78 (61.4) | 85 (66.9) | 12 (63.2) |
| 1–10% | 27 (44.3) | 31 (25.4) | 31 (24.4) | 18 (14.2) | 1 (5.3) |
| 11–20% | 3 (4.9) | 7 (5.7) | 9 (7.1) | 7 (5.5) | 1 (5.3) |
| >20% | 2 (3.3) | 5 (4.1) | 2 (1.6) | 9 (7.1) | 0 (0) |
| NE | 1 (1.6) | 0 (0) | 5 (3.9) | 3 (2.4) | 3 (15.8) |
| Missing | 2 (3.3) | 3 (2.5) | 2 (1.6) | 5 (3.9) | 2 (10.5) |
| Increased airway thickness |  |  |  |  |  |
| Present | 38 (62.3) | 102 (83.6) | 119 (93.7) | 120 (94.5) | 17 (89.5) |
| Absent | 21 (34.4) | 17 (13.9) | 5 (3.9) | 2 (1.6) | 0 (0) |
| NE | 0 (0) | 0 (0) | 1 (0.8) | 0 (0) | 0 (0) |
| Missing | 2 (3.3) | 3 (2.5) | 2 (1.6) | 5 (3.9) | 2 (10.5) |
| Paraseptal emphysema |  |  |  |  |  |
| Absent | 34 (55.7) | 32 (26.2) | 27 (21.3) | 25 (19.7) | 0 (0) |
| Mild | 20 (32.8) | 43 (35.2) | 41 (32.3) | 42 (33.1) | 3 (15.8) |
| Moderate to severe | 5 (8.2) | 44 (36.1) | 56 (44.1) | 55 (43.3) | 14 (73.7) |
| NE | 0 (0) | 0 (0) | 1 (0.8) | 0 (0) | 0 (0) |
| Missing | 2 (3.3) | 3 (2.5) | 2 (1.6) | 5 (3.9) | 2 (10.5) |

For soluble biomarkers in whole blood, P values are shown for comparison against ex-smokers without airflow limitation.

*A1ATD* alpha-1-antitrypsin deficiency, *CT* computed tomography, *EwAL* ex-smokers without airflow limitation, *GOLD* Global Initiative for Chronic Obstructive Lung Disease, *NE* not evaluable, *SD* standard deviation.

**Table S3.** DL_CO_ parameters

| **DL_CO_ parameters, mean (SD)** | **EwAL** | **GOLD 1** | **GOLD 2** | **GOLD 3** | **A1ATD** |
| --- | --- | --- | --- | --- | --- |
| DL_CO_ corrected for Hb, mmol/min/kPa | 7.15 (4.81) | 8.38 (5.44) | 5.89 (4.13) | 4.52 (3.24) | 3.23 (1. 23 |
| VA, mL | 6.1 (1.3) | 6.0 (1.1) | 5.4 (1.3) | 4.8 (1.4) | 6.1 (1.5) |
| DL_CO_/VA corrected for Hb, mmol/min/kPa | 1.18 (0.75) | 1.41 (0.86) | 1.13 (0.75) | 1.02 (0.75) | 0.52 (0.12) |

*A1ATD* alpha-1-antitrypsin deficiency, *DL_CO_* diffusing capacity of the lung for carbon monoxide, *EwAL* ex-smokers without airflow limitation, *GOLD* Global Initiative for Chronic Obstructive Lung Disease, *Hb* haemoglobin, *kPa* kilopascal, *mmol* millimole, *SD* standard deviation, *VA* alveolar volume.
